# GEAR: an integrated atlas of gene expression dynamics in *Arabidopsis thaliana*

**DOI:** 10.1093/nar/gkaf1056

**Published:** 2025-11-04

**Authors:** Motomu Endo, Masaki Ito, Akane Kubota, Nozomu Takahashi

**Affiliations:** Nara Institute of Science and Technology, Takayama-cho, Ikoma, Nara 630-0192, Japan; Nara Institute of Science and Technology, Takayama-cho, Ikoma, Nara 630-0192, Japan; Nara Institute of Science and Technology, Takayama-cho, Ikoma, Nara 630-0192, Japan; Nara Institute of Science and Technology, Takayama-cho, Ikoma, Nara 630-0192, Japan

## Abstract

Circadian rhythms regulate crucial physiological processes in plants, making the analysis of rhythmic gene expression vital for understanding how plants adapt to and anticipate daily environmental changes, and for improving agricultural traits. Foundational databases for plant circadian rhythms have been invaluable, but face challenges in keeping pace with the rapid accumulation of modern transcriptomic data, particularly for the most widely used model organism, *Arabidopsis thaliana*. To address this critical gap, we developed GEAR (Gene Expression Archives for Rhythm), a new database resource dedicated to *A. thaliana* that serves as an integrated atlas of gene expression dynamics. GEAR builds a comprehensive data archive by integrating both legacy microarray and modern RNA-seq datasets, covering 83 diverse experimental conditions. Its intuitive web interface functions as an interactive atlas, allowing users to compare expression profiles not only across different experimental treatments but also across independent studies conducted under nominally identical settings. This unique feature enables researchers to move beyond single-experiment findings and distinguish robust biological signals from interlaboratory variability. For the first time, this allows for a meta-analysis to define a high-confidence set of core clock-regulated genes, providing foundational insights into the most conserved outputs of the plant circadian network.

## Graphical Abstract

**Graphical Abstract:**
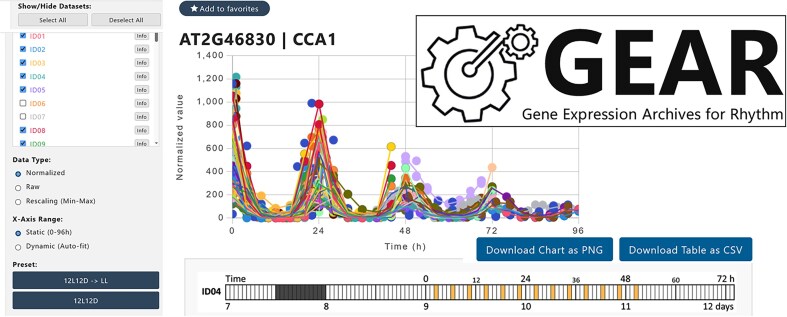

## Introduction

Circadian rhythms, intrinsic biological clocks with an ~24-h periodicity, govern a wide range of physiological processes in organisms [1, 2]. In the model organism *Arabidopsis thaliana*, these rhythms are fundamental to orchestrating growth and development [3–5], flowering time [6–8], and responses to environmental cues such as pathogens [9, 10]. The molecular basis of these rhythms relies on intricate transcriptional-translational feedback loops of core clock genes, which in turn drive the rhythmic expression of thousands of clock-controlled genes that influence cellular and systemic functions. A deeper understanding of these rhythmic processes is crucial not only for unraveling plant adaptation strategies but also for practical applications in agriculture, such as “chronoculture” [11, 12].

The advent of high-throughput omics has led to an exponential increase in publicly available time-series transcriptome data, making integrated databases essential for modern research. The DIURNAL database [13], developed as a pioneering resource, has long served as the de facto standard for the plant circadian community, making invaluable contributions. However, the landscape of genomics has evolved dramatically, and the rapid accumulation of modern RNA-seq data presents new challenges and opportunities. Existing resources, such as DIURNAL and CircaKB [14], have pursued “breadth” by enabling multi-species comparisons. While commendable, this approach can inherently limit the “depth” and timely updating for any single organism.

This situation has created a critical gap for researchers focused on *A. thaliana*. Although DIURNAL remains historically significant and has since expanded to include other plant species, its foundational dataset for *Arabidopsis* has not seen significant augmentation since its initial release. This means a vast trove of modern RNA-seq experiments remains scattered across general-purpose repositories. While these repositories are highly valuable as data sources for secondary analysis, they lack specialized interfaces for circadian biology, making it difficult to identify relevant time-series datasets or retrieve key experimental parameters without manually reviewing individual publications. Furthermore, older platforms can face technical challenges, such as a lack of modern security support, which can hinder reliable access. The need for an integrated database that allows for cross-dataset searching and utilization is a shared requirement, not only for the plant circadian rhythm community but also for researchers in related fields.

To address this need, we developed GEAR (Gene Expression Archives for Rhythm), a new database resource with a strategic focus on providing unparalleled depth and quality for the *A. thaliana* community. While discoveries in other species are invaluable, knowledge derived from *Arabidopsis* continues to form the bedrock of plant biology, making a comprehensive and up-to-date resource for this organism uniquely powerful. By unifying both legacy microarray and recent RNA-seq datasets in a single, cohesive platform, GEAR enables researchers for the first time to validate foundational findings and synthesize new knowledge across different technologies. The GEAR archive currently contains 83 diverse experimental conditions, representing a more than four-fold increase in dataset volume for *Arabidopsis* compared to its predecessors (Table 1).

**Table 1.** Comparison of features across plant circadian expression databases

Feature	GEAR (This work)	DIURNAL	CircaKB
Total experimental conditions(*Arabidopsis thaliana*)	83	20	6
*From public repositories*	76	17	6
*Unpublished*	7	3	0
Data type			
*Microarray*	30	20	3
*RNA-seq*	53	0	3
Unique genotype	17	5	1
Temporal coverage	2005–2024	2005, 2007, 2008	2014, 2018, 2021
Bulk data download	Yes	No	No

Presented as an interactive atlas, GEAR is also designed from the ground up to overcome critical usability limitations and accelerate research workflows. Previously, identifying specific experimental conditions required laborious searches through individual publications. GEAR replaces this with a powerful filtering system where users can find relevant datasets in just a few clicks using structured attributes like genotype and treatment. For deeper context, the platform provides direct hyperlinks for each gene to major external databases, including TAIR [15], KEGG [16], and Expression Atlas [17]. To facilitate large-scale meta-analyses and other secondary research, GEAR offers flexible options to download image files or processed data for individual experiments or for the entire dataset in bulk. By providing a robust, user-friendly, and continually updated resource, GEAR aims to accelerate new discoveries in plant circadian biology.

## Materials and methods

### Data collection and curation

Time-series gene expression data for inclusion in GEAR were primarily identified through extensive literature searches of the PubMed database, with subsequent data retrieval from public repositories, including the NCBI Gene Expression Omnibus (GEO) [18] and Sequence Read Archive (SRA) [19]. Datasets were selected based on criteria of having at least six sampling points per day and covering a minimum of 24 h. The current version of GEAR archives a total of 83 distinct experimental conditions, sourced from public data released between 2005 and 2024, alongside seven unpublished datasets from our laboratory. To ensure full transparency and allow for independent verification, these unpublished datasets have also been submitted to the GEO repository. The original source publications and repository accession numbers for each dataset are provided in Supplementary Table S1 [20–47]. Users can therefore access the original raw files (e.g. FASTQ, CEL) for all datasets to perform their own analyses.

### Data processing and normalization

The data processing pipeline was designed to unify expression values from disparate sources and technologies. The process consisted of two main stages: (i) initial processing to generate a baseline expression value for each dataset, and (ii) a global normalization step to ensure robust comparability across all 83 experimental conditions (see Supplementary data S1 for detailed protocols).

#### Initial processing of “raw” data

For microarray datasets, the starting point was the processed data as provided by the original authors in public repositories (e.g. GEO), which had typically undergone initial normalization such as RMA. For RNA-seq datasets, raw FASTQ files were processed using the RaNA-seq analysis platform (v1.3.3) [48]. While this platform offers a convenient workflow, the core quantification and per-experiment normalization were performed using its built-in pipeline based on the DESeq2 method [49], a de facto standard in transcriptomics. The collection of these 83 initially processed datasets constitutes the “raw” data view in GEAR.

#### Global normalization and data views

While the “raw” data is valuable, expression levels are not directly comparable across experiments due to differing platforms and normalization baselines. To address this, a global normalization was applied to the entire collection of 83 “raw” datasets (both microarray and RNA-seq). We employed the MBQN R package, which implements a tail-robust quantile normalization (TRQN) method [50]. Classical quantile normalization, while widely used, can introduce bias when applied to datasets containing features with consistently high or low expression across many samples, a characteristic common in large-scale transcriptomic atlases. Such features can have their biological variance artificially suppressed, impeding statistical inference. The TRQN method is specifically designed to overcome this limitation by preserving the biological signals of these features in the tails of the data distribution, making it a more suitable choice for generating a cohesive and reliable cross-experiment dataset for this atlas. This procedure generated the “normalized” data, which is used for cross-experiment comparison on the web interface. For visual comparison of waveform shapes, a “rescaled” view is also provided, where the “normalized” values for each gene are scaled to a 0–1 range.

### Rhythmicity analysis

To enhance the utility of the database, we performed a comprehensive rhythmicity analysis for all genes across all 83 experimental conditions. The widely used MetaCycle R package (v1.2.0) was employed to detect periodic expression patterns. For each gene in each dataset, rhythmic parameters, including period, phase, and *P*-value, were calculated using the default JTK_CYCLE algorithm within the MetaCycle suite. The results of this analysis (*P*-value, period, and relative amplitude) are displayed on the gene search result page, allowing users to immediately assess the statistical significance and characteristics of circadian rhythms under various conditions. This pre-computed analysis provides a powerful tool for hypothesis generation and for identifying robustly cycling genes.

### Database implementation and web interface

The GEAR backend is built on a relational database managed by MySQL, which stores all time-series data, metadata, and pre-computed rhythmicity parameters. The web interface is a custom application built with PHP server-side scripts that dynamically query the database and present the data. The frontend leverages the Chart.js JavaScript library to generate interactive and responsive visualizations of gene expression profiles. This user interface is designed for intuitive data exploration, allowing researchers to search for genes (e.g. by AGI code) and filter the 83 experimental conditions by key attributes such as ecotype, genotype, light, and temperature. Interactive features include the ability to visualize up to 10 genes simultaneously, toggle between data normalization types (raw, normalized, and rescaled), adjust the *X*-axis range (static versus dynamic), and access detailed dataset information and original publication links via an “Info” button.

### Data accessibility and future development

GEAR is publicly and freely accessible at https://scientist.xsrv.jp/ with no login requirements. All time-series data can be viewed online and downloaded for secondary analysis. To ensure long-term utility, we commit to maintaining GEAR for a minimum of five years post-publication and performing regular data updates every few months to incorporate new datasets from recent publications. Having implemented the core rhythm detection functionality, future development will focus on integrating more advanced analytical tools, such as co-expression network analysis and comparative visualization of circadian parameters (e.g. phase-enrichment plots) across multiple conditions. Instructions for researchers wishing to contribute their own datasets to GEAR are available on the website. Future expansion to include other key plant species is also planned.

## Results

### Design and organization of GEAR

GEAR was designed as a user-centric and interactive web-based atlas to explore the wealth of gene expression dynamics data in *A. thaliana*. The core architecture is built upon a flexible content management framework, meticulously designed for efficient data organization, intuitive browsing, and powerful visualization. The database is built upon a comprehensive data archive, containing 83 distinct experimental conditions that encompass a wide variety of genotypes and environmental stimuli (Table 1). The interface allows researchers to seamlessly navigate this large collection of data, overcoming the accessibility and usability limitations of previous resources.

### Powerful search and filtering capabilities

GEAR provides two primary, flexible avenues for users to locate and explore data. For gene-centric queries, users can input a gene identifier (e.g. AGI code) into the main search bar to retrieve all available time-series data for that specific gene across all 83 conditions.

**Figure 1.**
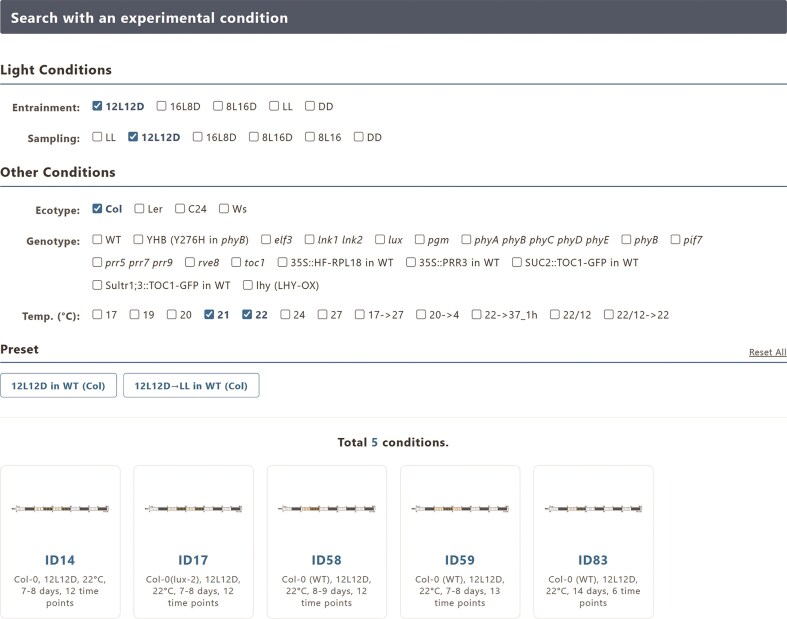
The condition-centric search interface of GEAR. Users can intuitively filter the entire collection of 83 experimental conditions by selecting biologically meaningful attributes from structured menus, such as light conditions, ecotype, genotype, and temperature. The example shows a search for wild-type (Col) experiments conducted at 21°C or 22°C under a 12h-light/12h-dark cycle, which yielded four matching conditions displayed at the bottom. Preset buttons for common queries are also provided for convenience.

Alternatively, for condition-centric exploration, GEAR features a powerful and intuitive filtering interface (Fig. 1). A major advancement over older, ID-based systems, this interface allows users to filter the entire collection of datasets using biologically meaningful attributes. Structured menus for Ecotype, Genotype, Light Conditions, and Temperature enable researchers to find relevant datasets in a few clicks. For example, a user can instantly find all datasets for the elf3 mutant under continuous light. For each dataset, detailed experimental protocols from the original publication are also directly accessible, eliminating the need for researchers to hunt for the source paper to understand the experimental context.

### Interactive visualization of gene expression patterns

Once relevant datasets are identified, GEAR provides a flexible platform to visualize gene expression profiles. The platform generates interactive plots using Chart.js, with numerous options for in-depth analysis.

**Figure 2.**
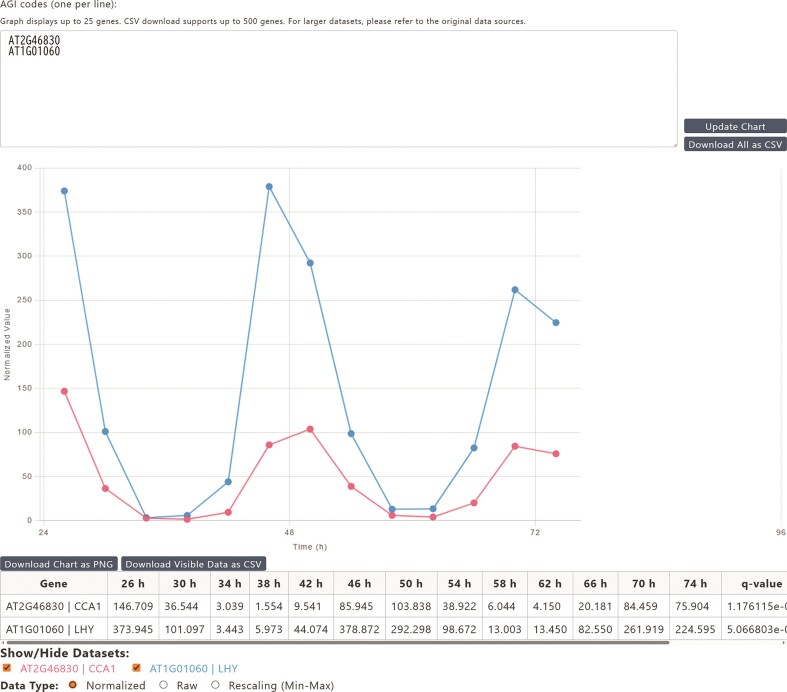
Comparative analysis of multiple gene expression profiles in GEAR. Users can input up to 25 gene identifiers (AGI codes) to simultaneously visualize their temporal expression patterns within a selected experimental condition. The example plot shows the normalized expression of several core clock genes (e.g. *LHY, CCA1, TOC1, PRR7, GI*, and *ELF4*), highlighting their characteristic, phased oscillations. An interactive legend below the plot allows individual gene profiles to be toggled on or off for clarity.

**Figure 3.**
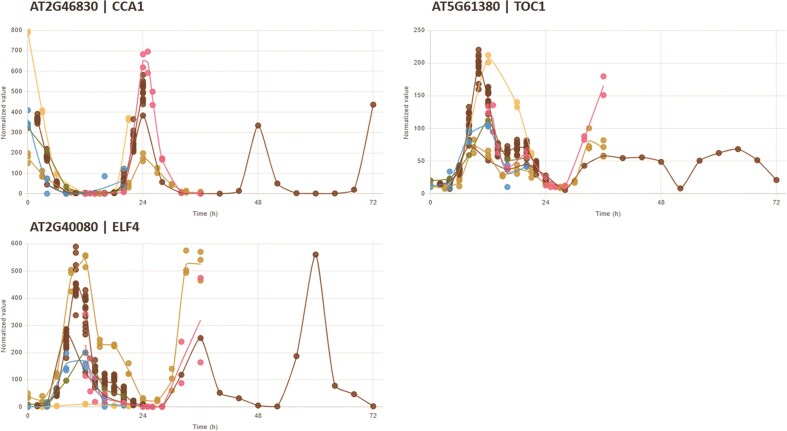
In-depth analysis of a single gene’s expression across multiple experimental conditions. GEAR can display the expression profile of a single gene from up to 25 different experimental conditions simultaneously, allowing researchers to assess the consistency of a gene’s rhythm. The figure shows representative plots for three core clock genes: (**A**) *CCA1*, (**B**) *TOC1*, and (**C**) *ELF4*. The interface provides interactive controls to toggle datasets and data types, and offers detailed gene descriptions with direct links to major external databases (e.g. TAIR, KEGG) for deeper biological context.

- Comparative analysis of multiple genes: Users can select one or more experimental conditions and input up to 25 distinct gene identifiers to generate a single plot displaying the temporal expression patterns of all selected genes (Fig. 2). After rendering, the visibility of each gene’s expression profile can be interactively toggled on or off, allowing for a clear, uncluttered view.
- In-depth analysis of a single gene across multiple conditions: Alternatively, users can focus on a single gene and compare its expression across up to 10 different experimental conditions (Fig. 3). This view is equipped with several powerful features. An interactive legend allows users to dynamically show or hide the expression profile for each condition. A single click allows toggling between normalized data (for comparing across experiments), raw data (for assessing absolute expression levels), and rescaled data (min–max normalization for visual waveform comparison). To balance comparability and clarity, the *X*-axis range can be switched between a fixed “static (0–96 h)” view and a “dynamic (Auto-fit)” view. For deeper biological context, direct hyperlinks to major external databases such as TAIR, KEGG, and Expression atlas are also provided.

### Comprehensive rhythmicity analysis

A key feature of GEAR is the pre-computed rhythmicity analysis for all genes across all 83 conditions. Using the MetaCycle JTK_CYCLE algorithm, we identified a substantial number of rhythmically expressed genes, providing a global overview of circadian regulation under diverse circumstances. The results, including *P*-value, period, and relative amplitude, are integrated directly into the gene search results, allowing users to rapidly identify candidate genes and formulate new hypotheses without the need to perform their own initial analysis.

### Application examples

To demonstrate the utility of GEAR as a hypothesis-generation platform, we conducted a meta-analysis of core rhythmic genes under free-running conditions.

- Case 1: Assessing the reproducibility of rhythmic gene identification. A common experiment in circadian biology involves entrainment under a 12 h-light/12 h-dark (12L12D) cycle followed by analysis in constant light (LL). We first used GEAR to identify seven independent datasets that followed this “12L12D→LL” protocol. A cross-dataset comparison revealed a surprisingly small overlap: of the thousands of genes identified as rhythmic in individual studies, only 437 genes were consistently rhythmic across all seven datasets, defined here as having a MetaCycle JTK_CYCLE *P*-value < .05 in each dataset (Table 2). This finding highlights significant inter-study variability and underscores the critical need for cross-dataset validation to define a high-confidence set of “truly” rhythmic genes.
- Case 2: Identifying core robustly regulated biological pathways. We next performed a GO-term enrichment analysis [51] on this high-confidence set of 437 “truly oscillating genes” to identify the biological processes most robustly controlled by the circadian clock. As expected, terms related to the “circadian rhythm” itself were highly enriched. Notably, the majority of the other top 20 enriched GO terms were strongly associated with photosynthesis and response to light stimulus (Table 2). This result demonstrates GEAR’s power not just to confirm known biology, but to reveal which pathways are the most robustly and consistently regulated outputs of the circadian system across variable experimental conditions.

**Table 2.** Gene Ontology (GO) term enrichment analysis of robustly rhythmic genes

GO-term enrichment by biological process	*P*-value
Response to light stimulus (GO:0009416)	1.27 × 10^–27^
Response to radiation (GO:0009314)	6.31 × 10^–27^
Circadian rhythm (GO:0007623)	1.26 × 10^–26^
Rhythmic process (GO:0048511)	1.26 × 10^–26^
Response to abiotic stimulus (GO:0009628)	3.69 × 10^–26^
Response to stimulus (GO:0050896)	1.40 × 10^–14^
Response to red or far red light (GO:0009639)	5.19 × 10^–14^
Regulation of circadian rhythm (GO:0042752)	3.68 × 10^–12^
Photosynthesis (GO:0015979)	2.09 × 10^–11^
Photosynthesis, light reaction (GO:0019684)	7.13 × 10^–11^
Response to temperature stimulus (GO:0009266)	7.42 × x10^-11^
Pigment biosynthetic process (GO:0046148)	2.26 × 10^–10^
Pigment metabolic process (GO:0042440)	1.43 × 10^–09^
Small molecule metabolic process (GO:0044281)	1.14 × 10^–08^
Flavonoid biosynthetic process (GO:0009813)	3.12 × 10^–08^
Photosynthesis, light harvesting in photosystem I (GO:0009768)	9.39 × 10^–08^
Photosynthesis, light harvesting (GO:0009765)	1.19 × 10^–07^
Chloroplast organization (GO:0009658)	1.25 × 10^–07^
Generation of precursor metabolites and energy (GO:0006091)	1.25 × 10^–07^
Plastid organization (GO:0009657)	1.27 × 10^–07^

The analysis was performed on a high-confidence set of 437 genes identified as rhythmically expressed across seven independent wild-type datasets under “12L12D→LL” conditions downloaded from GEAR. A gene was included in this set if it met the significance threshold (*P*-value < .05) in the MetaCycle JTK_CYCLE analysis for all seven datasets. The table shows the most significantly enriched terms for “Biological Process” and “Cellular Component,” ranked by *P*-value.

### Data accessibility and download

All data visualized in GEAR is readily accessible. Users can download publication-quality images of any graph. To facilitate the secondary analyses described in Case 2, the underlying raw numerical data for each experiment can be downloaded individually. Furthermore, a bulk-download option is available from the top page, enabling users to efficiently retrieve the entire collection of datasets. For more targeted queries, users can download expression data as a CSV file for up to 500 genes at once.

## Discussion

We have developed GEAR, a comprehensive and interactive atlas of gene expression dynamics in *A. thaliana*. This work was motivated by a critical need within the plant science community for a modern, reliable, and deep resource for circadian gene expression. GEAR directly addresses the limitations of previous platforms by massively expanding the data volume, integrating both legacy and modern technologies, and providing a powerful, user-centric interface.

A key contribution of this work is not to report a single biological finding, but to provide a foundational research infrastructure that accelerates future discoveries. By enabling robust cross-experiment comparisons, GEAR allows researchers to move beyond the limitations of individual studies. As demonstrated in our case study, the ability to identify a high-confidence set of “truly” rhythmic genes across multiple independent experiments is a powerful approach. The finding that photosynthesis-related pathways are among the most robustly regulated outputs of the clock provides a new layer of insight into the hierarchical organization of the circadian network—a conclusion that would be difficult to draw from any single dataset.

Our initial development of GEAR has been deliberately focused on *A. thaliana*. This was a strategic decision to provide unparalleled “depth” and quality for the community’s most-used model organism, where the demand for a modern data resource is most acute. This approach contrasts with, and complements, other resources that aim for “breadth” across many species. The database architecture was, however, designed from the outset to be fully scalable for future expansion.

Of course, GEAR has its limitations. The quality and resolution of the data are inherently dependent on the original studies. While our unified pipeline and tail-robust normalization mitigate some variability, users should consider the original technology when interpreting results. Having now implemented a comprehensive rhythmicity analysis, future development will focus on integrating more advanced analytical functionalities, such as co-expression network analysis and comparative visualization of circadian parameters across conditions.

In conclusion, GEAR represents a significant leap forward for the plant circadian research field. By providing a comprehensive, user-friendly, and continually updated data resource with a clear commitment to long-term maintenance and built-in analytical capabilities, GEAR effectively revitalizes the research infrastructure for studying gene expression dynamics. We anticipate that GEAR will become an indispensable tool, fostering deeper insights into the complex interplay between the circadian clock and plant physiology.

## Supplementary Material

gkaf1056_Supplemental_File

## Data Availability

GEAR is publicly and freely available at https://scientist.xsrv.jp/. To ensure long-term preservation and stable accessibility beyond the lifetime of the primary website, the complete raw data collection described in this study has also been deposited in the Zenodo repository and is citable via the DOI: 10.5281/zenodo.15910335. The detailed metadata for all datasets, including source accession numbers and original publication links, are available inSupplementary Table S1.

## Notes

Present address: Division of Biological Science, Nara Institute of Science and Technology,Ikoma, Nara 630-0192, Japan
