# Supplementary material for: GEAR: an integrated atlas of gene expression dynamics in Arabidopsis thaliana
Source: Nucleic Acids Res. 2025 Nov 4;54(D1):D1711–9. doi: 10.1093/nar/gkaf1056 (PMC12807683; doi:10.1093/nar/gkaf1056)
Supplement: gkaf1056_Supplemental_File [file gkaf1056_supplemental_file.pdf]

Supplementary Table S1: Detailed Metadata of All Experimental Conditions in GEAR.

ID	Release on	Accession number	Platform	Ecotype	Genotype	Light condition	Light intensity	Temperature	Age	Tissue/Organ	Reference
1	2005/2/16	PRJNA100637	Affymetrix Arabidopsis ATH1 genome array	Col-0	Wild type	12L12D	150	20	29	Leaves	(22)
2	2005/12/1	PRJNA93443	Affymetrix Arabidopsis ATH1 genome array	Col-0	Wild type	12L12D	130	20	35	Leaves	(23)
3				Col-0	*pgm*	12L12D	130	20	35	Leaves	
4	2007/1/8	PRJNA96903	Affymetrix Arabidopsis ATH1 genome array	Col-0	Wild type	12L12D → LL	60-65	22	8-9	Whole seedlings	(24)
5	2007/7/15	PRJNA101389	Affymetrix Arabidopsis ATH1 genome array	Col-0	Wild type (CCR2::LUC)	12L12D → LL	-120	22	9-10	Whole seedlings	(25)
6	2007/9/18	E-TABM-331	Custom oligonucleotide microarrays	Col-0	Wild type	12L12D → LL	150	20	28	Leaves	(26)
7	2008/1/18	E-MEXP-1304	Affymetrix Arabidopsis ATH1 genome array	Col-0	Wild type	DD	0	22/12 → 22	7-8	Whole seedlings	(27)
8				Col-0	Wild type	12L1D	100	22/12	8-9	Whole seedlings	
9				Col-0	Wild type	LL	100	22/12	8-9	Whole seedlings	
10				Col-0	Wild type	12L12D → LL	100	22/12 → 22	9-10	Whole seedlings	
11				Col-0	Wild type	LL	100	22/12 → 22	9-10	Whole seedlings	
12				L*er*	Wild type	16L8D	90	22	8-9	Whole seedlings	
13				L*er*	Wild type	8L16D	180	22	8-9	Whole seedlings	
14	2008/10/17	E-MEXP-1299	Affymetrix Arabidopsis ATH1 genome array	Col-0	Wild type	12L12D	100	22	8-9	Whole seedlings	(28)
15				Col-0	Wild type	8L16D	100	22	8-9	Whole seedlings	
16				Ler	Wild type	8L16D	100	22	8-9	Whole seedlings	
17				Col-0	*lux-2*	12L12D	100	22	8-9	Whole seedlings	
18				Col-0	*phyB-9*	8L16D	100	22	8-9	Whole seedlings	
19				Ler	*lhy* (LHY OX)	8L16D	100	22	8-9	Whole seedlings	
20	2009/12/10	PRJNA120947	Affymetrix Arabidopsis ATH1 genome array	C24	Wild type	12L12D → LL	70 → 100	19	14-15	Aerial tissue	(29)
21				C24	Wild type + Nicotinamide	12L12D → LL	70 → 100	19	14-15	Aerial tissue	
22				C24	*toc1-1*	12L12D → LL	70 → 100	19	14-15	Aerial tissue	
23	2010/11/30	E-MEXP-2526	Affymetrix Arabidopsis ATH1 genome array	Col-0	Wild type	16L8D	150	20	40-45	Leaves	(30)
24				Col-0	Wild type	16L8D	150 → 90	20 → 4	40-45	Leaves	
25				Col-0	Wild type	16L8D → LL	150	20	40-45	Leaves	
26	2012/12/20	PRJNA159253	AGRONOMICS1 Arabidopsis tiling array	Col-0	Wild type	12L12D → LL	50-60	22	11-12	Whole seedlings	(31)
27				Col-0	*rve8-1*	12L12D → LL	50-60	22	11-12	Whole seedlings	
28	2013/8/2	PRJNA188075	Illumina Genome Analyzer Iix	Col-0	Wild type	16L8D	-	22	9	Whole seedlings	(32)
29				Col-0	*link1 link2*	16L8D	-	22	9	Whole seedlings	
30	2014/9/10	PRJNA217497	Affymetrix Arabidopsis Gene 1.0 ST Array	Col-0	Wild type	16L8D	56	22	9-10	Whole seedlings	(33)
31				Col-0	Wild type	16L8D	56	22	9-10	Whole seedlings	
32				Col-0	Wild type	16L8D	56	22	9-10	Whole seedlings	
33				Col-0	Wild type	8L16D	84	22	9-10	Whole seedlings	
34				Col-0	Wild type	8L16D	84	22	9-10	Whole seedlings	
35				Col-0	Wild type	8L16D	84	22	9-10	Whole seedlings	
36	2014/4/23	PRJNA245231	Illumina HiSeq 2000	Col-0	Wild type + Coronatine	16L8D	100	22/18	21-28	Whole seedlings	(34)
37				Col-0	Wild type	16L8D	100	22/18	21-28	Whole seedlings	
38	2015/10/16	PRJNA297861	Illumina HiSeq 1500	Col-0	Wild type	12L12D → LL	60-100	22	21	Shoot apex	(35)
39	2016/10/27	(SUB1572336)	Illumina HiSeq 2000	L*er*	WT	8L16D	170*	22	11	Ground seedlings	(36)
40				L*er*	WT	8L16D	170*	22	11	Ground seedlings	
41				L*er*	*phyA phyB phyC phyD phyE*	8L16D	170*	22	11	Ground seedlings	
42				L*er*	*phyA phyB phyC phyD phyE*	8L16D	170*	22	11	Ground seedlings	
43				L*er*	YHB (Y276H mutation in phyB)	8L16D	170*	22	11	Ground seedlings	
44				L*er*	YHB (Y276H mutation in phyB)	8L16D	170*	22	11	Ground seedlings	
45	2017/6/26	PRJNA384110	Illumina HiSeq 2000	Col-0	WT	8L16D	170	27	7	Whole seedlings	(37)
46				Col-0	WT	8L16D	170	22	7	Whole seedlings	
47				Col-0	*elf3-1*	8L16D	170	27	7	Whole seedlings	
48				Col-0	*elf3-1*	8L16D	170	22	7	Whole seedlings	
49				Col-0	*lux-4*	8L16D	170	27	7	Whole seedlings	
50				Col-0	*lux-4*	8L16D	170	22	7	Whole seedlings	
51	2017/11/9	PRJNA1291941	Illumina NextSeq 500	Col-0	WT	16L8D	45	22	10	Whole seedlings	Unpublished
52				Col-0	WT	8L16D	67-70	22	10	Whole seedlings	
53				Col-0	*SUC2::TOC1-GFP*	16L8D	45	22	10	Whole seedlings	
54				Col-0	*SUC2::TOC1-GFP*	8L16D	67-70	22	10	Whole seedlings	
55				Col-0	*Sultr1;3::TOC1-GFP*	16L8D	45	22	10	Whole seedlings	
56				Col-0	*Sultr1;3::TOC1-GFP*	8L16D	67-70	22	10	Whole seedlings	
57	2018/7/12	PRJDB5719	Illumina HiSeq 2000	Col-0	WT	12L12D → LL	100 → 180-200	22	16-17	Whole aerial part	(38)
58	2018/12/6	PRJNA475542	Illumina NextSeq 500	Col-0	WT	12L12D	170	22	7	Whole seedlings	(39)
59	2019/8/11	PRJNA559744	Illumina HiSeq 2500	Col-0	WT [PAT-seq]	12L12D	150	22	7	Whole seedlings	(40)
60	2020/1/14	PRJNA566413	Illumina HiSeq 4000	Col-0	WT	16L8D → LL	-	24	10	Whole seedlings	(41)
61				Col-0	WT	16L8D	-	24	10	Whole seedlings	
62	2020/4/14	PRJEB32446	Illumina HiSeq 2500	Col-0	WT	12L12D → LL	50	22	13-14	Whole seedlings	(42)

63	2020/6/2	PRJNA510535	Illumina NextSeq 500	Col-0	WT	16L8D	35-40	17	7	Whole seedlings	(43)
64				Col-0	WT	16L8D	35-40	17 → 27	7	Whole seedlings	
65				Col-0	*pif7-1*	16L8D	35-40	17	7	Whole seedlings	
66				Col-0	*pif7-1*	16L8D	35-40	17 → 27	7	Whole seedlings	
67	2021/4/21	PRJNA665236	Illumina NextSeq 500	Col-0	WT (*35S:HF-RPL18*)	12L12D → LL	130 → 80-100	22	13	Whole seedlings	(44)
68				Col-0	WT (*35S:HF-RPL18*) [TRAP-se	12L12D → LL	130 → 80-100	22	13	Whole seedlings	
69				Col-0	WT (*35S:HF-RPL18*)	12L12D → LL	130 → 80-100	22 → 37_1h	13	Whole seedlings	
70				Col-0	WT (*35S:HF-RPL18*) [TRAP-se	12L12D → LL	130 → 80-100	22 → 37_1h	13	Whole seedlings	
71	2023/12/5	PRJNA1043887	Illumina NovaSeq 6000	Col-0	WT	16L8D	100	22	21	3rd and 4th rosette leaves	(45)
72				Col-0	WT	16L8D	100	22	35	3rd and 4th rosette leaves	
73	2025/3/24	PRJCA021408	Illumina	Col-0	WT [scRNA-seq]	12L12D → LL	100	22	9	Seedling	(46)
74				Col-0	WT [scRNA-seq]	12L12D → LL	100	22	9	Seedling	
75	2024/1/29	PRJNA1291957	Illumina NextSeq 500	Col-0	WT	12L12D → LL		22	16	Whole seedlings	Unpublished
76				Col-0	*35S::+D77:G78PRR3*	12L12D → LL		22	16	Whole seedlings	
77				Col-0	*prr9 prr7 prr5*	12L12D → LL		22	16	Whole seedlings	
78	2024/8/26	PRJNA996904	Illumina NextSeq 500	Col-0	WT	12L12D	-100	20	11	Whole seedlings	(47)
79				Col-0	WT	12L12D → LL	-100	20	11	Whole seedlings	
80	2024/12/21	PRJNA1015621	Illumina NovaSeq 6000	Ws-2	WT	8L16D → 16L8D	70	21	12	3rd and 4th rosette leaves	(48)
81				Ws-2	WT	8L16D → 16L8D	70	21	12	3rd and 4th rosette leaves	
82				Ws-2	WT	8L16D → 16L8D	70	21	12	3rd and 4th rosette leaves	
83	2024/3/21	PRJDB16373	Illumina HiSeq 2500	Col-0	WT	12L12D	70	22	14	Whole shoot	(49)

A comprehensive list of all 83 experimental conditions, detailing for each entry its GEAR ID, source publication (PMID/DOI), public repository accession numbers (GEO/SRA) (including those for the seven previously unpublished datasets), and key experimental parameters including Arabidopsis thaliana ecotype, genotype, light and temperature conditions, sampling details, and tissue sampled. This information ensures full transparency and allows researchers to trace the origin of each dataset.

Supplementary Text S1: Data Processing and Normalization Pipelines

Overview

The primary goal of the GEAR data processing pipeline is to integrate time-series transcriptome data from diverse technologies (microarray and RNA-seq) and various experimental sources into a single, cohesive resource. The pipeline is designed to enable robust cross-experiment comparisons while preserving meaningful biological variance. The process involves two main stages: (i) initial processing of microarray and RNA-seq data to create a baseline "Raw" dataset, and (ii) a global normalization of all datasets to produce a comparable "Normalized" dataset for visualization and analysis.

Initial Data Processing

Microarray Data The starting point for microarray data was the processed expression values as provided by the original authors in the NCBI Gene Expression Omnibus (GEO) repository. These data were typically derived from raw CEL files and had already undergone an initial normalization step such as Robust Multi-array Average (RMA) or GC-RMA by the depositors. No re-processing from raw CEL files was performed. This collection of author-processed data constitutes the microarray portion of the "Raw" data pool in GEAR.

RNA-seq Data For RNA-seq experiments, raw data were retrieved as FASTQ files from the Sequence Read Archive (SRA). These files were then uploaded to the RaNA-seq analysis platform (v1.3.3). This platform was chosen for its user-friendly graphical interface and its efficient, end-to-end workflow, which allows for consistent processing of multiple datasets without requiring deep command-line expertise. The built-in pipeline, which utilizes the DESeq2 method for normalization within each experiment, was applied using the

platform's default parameters (Test = Wald, pValue cutoff = 0.05, Fit Type = parametric). The resulting expression datasets form the RNA-seq portion of the "Raw" data pool in GEAR.

Global Normalization and Data Views in GEAR

The GEAR web interface provides three distinct data views—"Raw," "Normalized," and "Rescaled"—which correspond to different stages of the global normalization pipeline.

- **Raw Data** This view presents the data after the initial processing stage described above. It is a collection of 83 datasets where each dataset has been normalized *within* its original experimental context. At this stage, a direct comparison of expression values across different experiments is challenging due to variations in experimental platforms and dynamic ranges.
- **Normalized Data** To enable robust cross-experiment comparisons, all 83 "Raw" datasets (both microarray and RNA-seq) were subjected to a second, global normalization procedure using the

MBQN (Mean/Median based Quantile Normalization) R package. This method is a "tail-robust" modification of classical quantile normalization, specifically designed to preserve the biological variance of features that are consistently found in the tails of the expression distribution (i.e., always very high or very low). Unlike traditional methods that can artificially compress the variance of such features, MBQN employs a two-step process of offset subtraction and back-transformation to ensure that biologically relevant signals are maintained. The normalization was executed using the following parameters: mbqn.mtx <- mbqnNRI(mtx, FUN="median", na.rm=TRUE, low_thr=0.5, verbose=FALSE). The resulting dataset allows for direct and reliable comparison of gene expression levels across all conditions in GEAR.

- **Rescaled Data** This view is intended for the visual comparison of waveform shapes, independent of the absolute expression level. The Normalized data are

further transformed by scaling the expression values for each gene to a range of 0 (minimum expression) to 1 (maximum expression) across the time course.
